# System for Context-Specific Visualization of Clinical Practice Guidelines (GuLiNav): Concept and Software Implementation

**DOI:** 10.2196/28013

**Published:** 2022-06-22

**Authors:** Jonas Fortmann, Marlene Lutz, Cord Spreckelsen

**Affiliations:** 1 Institute of Medical Informatics Medical Faculty Rheinisch-Westfälische Technische Hochschule Aachen University Aachen Germany; 2 Smart Medical Technology for Healthcare Consortium of the German Medical Informatics Initiative Leipzig Germany; 3 Institute of Medical Statistics, Computer and Data Sciences Jena University Hospital Jena Germany

**Keywords:** clinical practice guideline, clinical decision support system, decision support techniques, computer-assisted decision making, guideline representation, workflow control patterns, workflow, clinical, decision making, support systems, software, eHealth, electronic health

## Abstract

**Background:**

Clinical decision support systems often adopt and operationalize existing clinical practice guidelines leading to higher guideline availability, increased guideline adherence, and data integration. Most of these systems use an internal state-based model of a clinical practice guideline to derive recommendations but do not provide the user with comprehensive insight into the model.

**Objective:**

Here we present a novel approach based on dynamic guideline visualization that incorporates the individual patient’s current treatment context.

**Methods:**

We derived multiple requirements to be fulfilled by such an enhanced guideline visualization. Using business process and model notation as the representation format for computer-interpretable guidelines, a combination of graph-based representation and logical inferences is adopted for guideline processing. A context-specific guideline visualization is inferred using a business rules engine.

**Results:**

We implemented and piloted an algorithmic approach for guideline interpretation and processing. As a result of this interpretation, a context-specific guideline is derived and visualized. Our implementation can be used as a software library but also provides a representational state transfer interface. Spring, Camunda, and Drools served as the main frameworks for implementation. A formative usability evaluation of a demonstrator tool that uses the visualization yielded high acceptance among clinicians.

**Conclusions:**

The novel guideline processing and visualization concept proved to be technically feasible. The approach addresses known problems of guideline-based clinical decision support systems. Further research is necessary to evaluate the applicability of the approach in specific medical use cases.

## Introduction

### Clinical Practice Guidelines and Computerized Decision Support

Quality of care benefits from the application of clinical practice guidelines (CPGs) [1,2]. Various guideline-based clinical decision support systems (CDSSs) have been designed and implemented in the past [3]. Such CDSSs have been shown to improve adherence to CPGs, thus potentially increase quality of care [4].

Most guideline-based support systems are designed to modify the routine clinical workflow in a way that requires the clinician to interact with the CDSS directly at specific points in time to obtain additional information or other kinds of guidance with respect to the current treatment. These systems usually encompass a model of the underlying clinical pathway of some intervention and locally intervene at specific steps to provide additional information or recommendations.

Even though the respective research spans several decades and encompasses various CDSS implementations of different shapes, CDSSs are only recently becoming a common part of clinical practice. Several reviews addressed potential factors influencing the success or failure of guideline-based CDSS [5-8]. Among others, insufficient understanding of the underlying clinical processes turned out to be a relevant factor. Most CDSSs interact with the user by providing recommendations, reminders, or notifications to be considered at the specific point in time when they are shown. Greenes et al [5] propose that this focus of the interaction of the CDSS with individual decisions and actions should be complemented by a perspective that also considers the entire workflow. We also noticed the need for more attention to the overarching process when participating in the development of a guideline-based CDSS to assist in the treatment of bloodstream infections [9]. In this medical use case, the clinical workflow is executed over a relatively long period of time (up to 10 days). Thus, the impact of point-in-time decisions on the future workflow is of particular importance.

Internally, most systems contain computerized CPG knowledge, but they do not reveal it to the physician as a timewise longitudinal view of a CPGs intended treatment process. Only the current decision is shown and justified. Information about how this decision will influence the future clinical workflows is not presented (nor how past decisions have impacted the clinical workflow until now).

On one hand, the focus on the current decision to be made or the next steps to be taken is prioritized over the long-term perspective. On the other hand, visualizing the entire guideline (often hundreds of pages of condensed information) would understandably lead to an unacceptable cognitive load.

In this article, we present GuideLine Navigator (GuLiNav), an approach that addresses this shortcoming by generating context-specific versions of otherwise static clinical workflow representations. GuLiNav does so by avoiding a strict distinction between the design view of a computer-interpretable guideline (CIG; global representation of the guideline structure) and the view of the guideline during execution (presentation of the actual state/user interaction at a certain point in the patient journey), which is prevalent in most previous approaches.

The context-specific guideline generated by GuLiNav represents a feasible compromise between reducing the clinician’s cognitive load (by reducing the information load presented to a manageable amount) and providing an overview of the global treatment situation (by preserving context-specific relevant information). It is meant to support the autonomy of the clinician by providing guideline-based advice that has been tailored to fit the context-specific circumstances.

### State of the Art

Apart from CIG-related research, previous results in the area of presentation of treatment histories and timelines are also related to the system we present. For example, Plaisant et al [10] developed the LifeLines system, which intends to visualize personal histories of individuals and as such can also be used in the medical domain. It starts with an overview of the entire history and facilitates zooming in and out to reveal information on various levels of granularity. This kind of research lays a foundation for adequate digital representation of personal timelines (from a human-computer interaction perspective). More recently, several newer approaches to the visualization of time-oriented clinical data specifically have been developed. The general motivation is to enhance the presentation of raw clinical data by using a knowledge base to apply medical knowledge for deriving patient-specific advice or abstract medical concepts from clinical data (eg, Shahar et al [11], Martins et al [12], and Klimov et al [13] have developed different visualization approaches).

Within the last decades, various proprietary formats for the computerized representation of CPGs have evolved. They can be classified into 3 groups: document models, decision trees/probabilistic models, and task-network models (TNMs) [14]. The most prominent category with respect to its use in the context of clinical decision support are TNMs. Various TNMs were designed, such as the GuideLine Interchange Format, Version 3 (GLIF3), SAGE, GASTON framework, GLARE system, HELEN framework, PROforma formal knowledge representation language, Asbru, and more [15-21].

Even though the specific details vary, the basic idea is that in contrast to unstructured (ie, not machine readable) CPGs published by medical expert boards around the world, computer-interpretable formats facilitate the implementation of CDSSs by providing a structured, interchangeable, and, most importantly, computer-interpretable definition of a clinical guideline. This is achieved by representing the guideline in the form of some sequence of clearly defined tasks, actions, and decisions. During the treatment of a patient, patient-specific data can then be applied to the CIG to create an execution instance of the CIG and provide patient-specific advice.

**Figure 1:**
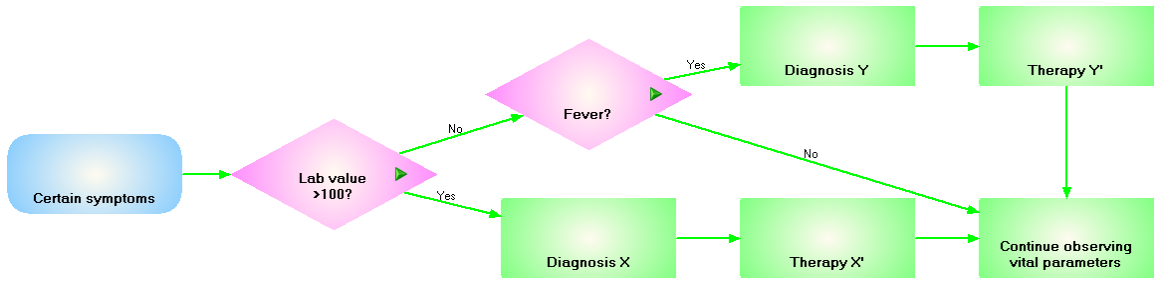
An example computer-interpretable guideline modeled in GuideLine Interchange Format (GLIF) using the GLIF Editor from the Medical Objects Knowledgebase.

For example, GLIF3 [15] uses 3 distinct levels to represent a CIG: conceptual, computable, and implementable. The conceptual level is represented as a flowchart (using a Unified Modeling Language [UML] Class Diagram) and acts as a structured documentation of the computerized guideline. Figure 1 shows the conceptual representation of an (imaginary) CIG. The computable level has its own syntax and defines the data, actions, and algorithm flow, and at the implementable level, a GLIF-based CIG can be incorporated in a specific health information system.

As another example, Asbru [21] uses a specialized syntax to specify so-called skeletal plans. Skeletal plans are, in short, temporal plans of procedures and actions captured on different levels of granularity. However, the Asbru syntax itself is defined in Backus-Naur form and thus difficult to read, even for domain experts. Thus, to visualize Asbru-based CIGs, AsbruView [22] was developed. It is a multidimensional representation that uses a traffic metaphor with different tracks representing different plans. The different axis then represents parallel plans or a decomposition of the underlying plan on a different level of detail.

In recent years, the notion that most of these specialized attempts are based on an equivalent foundation and can be, more or less, mapped from one to another, has emerged. This notion has surfaced outside of the medical domain: Russel et al [23] formally defined recurring patterns of workflows (using Petri Nets) and showed that the most prominent workflow systems, process models, and other related technical standard formats (such as SAP Workflow, FileNet, BPMN, UML 2.0 Activity Diagram, event-driven process chains) all express a large subset of the formally defined workflow control patterns and thus are, more or less, equivalent to each other.

Consequently, Mulyar et al [24] analyzed in which degree CIG representation can also be reduced to the same workflow control patterns. Despite the fact that a clinical guideline is inherently different from business workflows in the sense that it focuses on a single entity (the patient), they showed that CIGs also consist of basic workflow control patterns. They especially concluded that business process and model notation (BPMN), a prominent workflow modeling language from the business domain, has an equivalent expressiveness to the more specialized CIG formats.

More recently, guideline-based CDSSs have been developed using BPMN [25], often combined with ARDEN Syntax [26] modules, for the definition of the computerized guideline model. For example, de Bruin et al [27] used a combination of BPMN and ARDEN Syntax to model a clinical guideline for the prevention of transmission of hepatitis B from the mother to the newborn child. As another example, Rodriguez-Loya et al [28] used a combination of BPMN and a rule engine to diagnose chronic obstructive pulmonary disease as part of a workflow.

The computerized guideline representations are mostly intended as technical components to facilitate the implementation of guideline-based CDSSs but have no impact on what the actual end user of the CDSS sees.

Past guideline representation approaches chose between 2 extremes: not taking the treatment context into consideration at all (eg, the guideline document itself or certain static pathways derived from it) or only presenting isolated single point-in-time decisions to the user without providing an overview of the workflow before and after that point in time. The first variant also presents information that is irrelevant with respect to a specific context (information overload) and consequentially also leads to a high cognitive load for the user. The second variant does not embed the current options into the larger scope of a treatment (what happened, what could happen in the future) and delegates these considerations to the user themself, who has to memorize this information. As a consequence, this kind of information underload also leads to a high cognitive load for the user. GuLiNav’s contextualized guideline approach compromises between these 2 extremes.

## Methods

### Guideline Representation and Visualization

**Figure 2:**
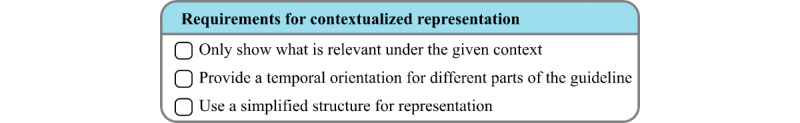
Short checklist of the requirements for contextualized representation.

Clinical pathways are complex to define, but when being presented to humans, a schematic description usually suffices since humans are capable of filling in the information gaps using their implicit knowledge. However, the complexity increases significantly when the need for operationalization arises and every detail needs to be specified explicitly (compare, for example, with the qualitative study about lessons learned when implementing clinical decision support by Wright et al [29]). This makes it especially difficult to find a visual representation that is entirely machine readable and still somehow accessible to humans. We have identified 3 aspects for human-readable guideline visualization (see Figure 2) that we will explain in more detail in the further course of this article.

**Figure 3:**
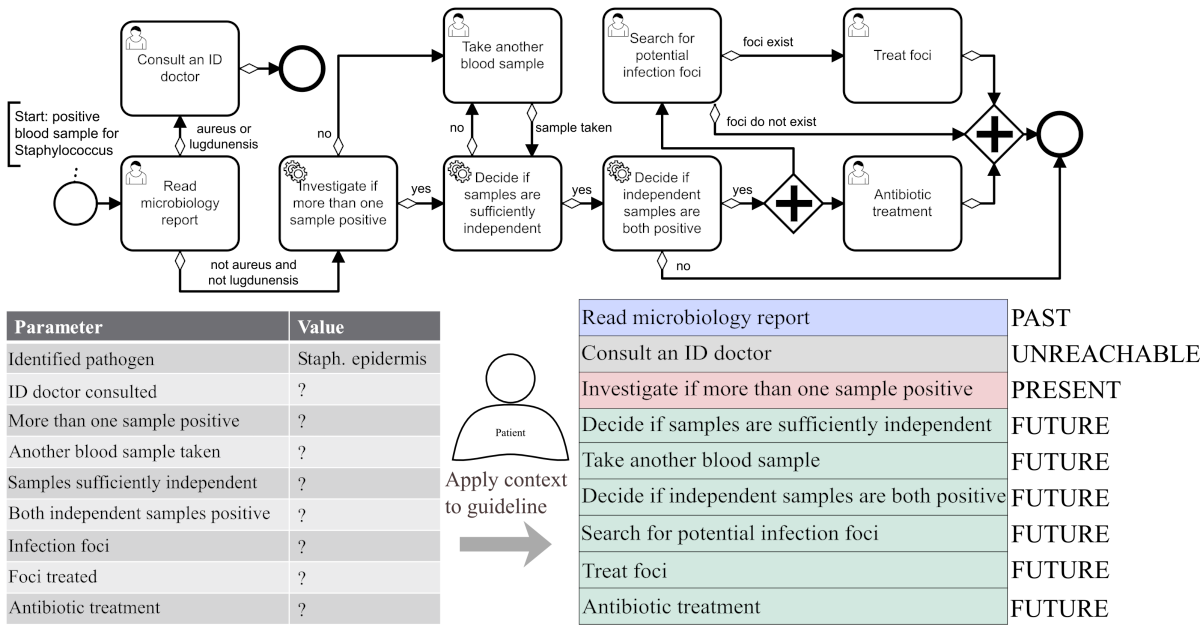
Context-based guideline visualization—Overview: Given a guideline definition and a treatment context (left), a context-sensitive guideline representation is generated (right).

**Figure 4:**
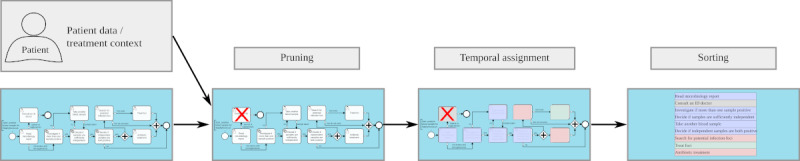
Context-based guideline visualization—Processing steps: Intermediate results after each processing step during the generation of a context-sensitive guideline.

We chose to use BPMN as a visual specification that represents the procedural aspects of the pathway (guideline procedures layer), while the medical criteria can be specified in an arbitrary fashion (eg, hard-coded, ARDEN Syntax...; medical criteria layer). This 2-way split of the guideline representation is a concept further explained and justified in Fortmann and Spreckelsen [30]. Other authors have suggested similar variants of separation between these two aspects (eg, Shahar et al [31] or Hatsek et al [32] suggest similar options on how to structure a computerized guideline). The decision to use BPMN is motivated by the fact that it can be used for the representation of CPGs [23,24,33], and there are also multiple useful tools, frameworks, and other resources freely available to work with it. Examples of these guidelines can be seen in Figure 3 and Figure 4. The examples are adaptations of the guideline used within the Hospital-Wide Electronic Medical Record Evaluated Computerized Decision Support System to Improve Outcomes of Patients With Staphylococcal Bloodstream Infection (HELP) study for the treatment of staphylococcal bloodstream infections [9], which initially motivated this research. Note that the example is simplified to be a fitting example for the rather technical focus of this article. The procedural view (visualized in BPMN) should be dynamized by taking an individual treatment context into consideration.

### Ethics Approval

The project uses only synthetic, generated data for testing. At no point in time was any real patient data used. The evaluation was done with nonpatients (colleagues from the university hospital). In similar former cases, especially evaluation studies including participants from the university (students, staff), the ethical committee of RWTH Aachen University Hospital was consulted and showed no apprehension about the studies. It declared that its consent was not required (eg, Ethical IDs: 2019: 269/18, 2020: 270/18).

### Context-Specific Guideline Visualization

#### Identification of 3 Aspects for Concept-Specific Visualization

Here we use the concept of a context-specific visualization. By context-specific we mean that clinical and patient data from a given situation are considered algorithmically to generate a guideline visualization adapted to the respective circumstances.

We identified 3 aspects where the context-specific visualization of a guideline should exploit context information to make the visualization more applicable (see Figure 2). Furthermore, we specified how each of these aspects can be considered when generating the context-sensitive guideline representation. As described in the introduction, the overarching goal is the prevention of cognitive overload by shifting away from decision support for point-in-time decisions (to prevent information underload). Instead, we want decision support for perspective decisions that underlines the entire treatment history and points to possible directions for treatment while eliminating irrelevant information (to prevent information overload).

#### Show Only What Is Relevant Under the Given Context (Pruning)

Pathways usually contain forks that, depending on some external information, indicate in which direction the flow will continue. These kinds of if-then-else conditions usually cause entire parts of a pathway to become irrelevant once all information needed to make the decision is available. Obviously, this information can change and reevaluation is necessary whenever we see a change in relevant data. Thus, the unreachable part of the static definition should be conditionally pruned and not shown in the contextualized representation.

#### Provide a Temporal Orientation for Different Parts of the Guideline (Temporal Assignment)

During different points in time during the execution of a clinical pathway, specific tasks are performed in a certain order. Generally, there can be more than one formally correct order and more than one task can be active at the same time. In addition, the same task can be active multiple times (circles). Nonetheless, we can assign a temporal role to each task that describes if the task was already executed (and should not be executed again; PAST) or is a choice that can currently be chosen (PRESENT) or might possibly be executed at a later time (FUTURE). Thus, the temporal role of each task should be visualized (ie, by assigning a color to each temporal role and then coloring each task respectively). Note that the temporal role PAST is only assigned if the task cannot occur again, and the temporal role FUTURE is also assigned to tasks that were already executed if they can potentially be executed again in the future.

#### Simplify the Structure for Representation (Topological Sorting)

Since only relevant parts of the pathway are shown and other branches of the pathway are pruned, the context-sensitive pathway already has a simpler structure. However, the structure should be further simplified to reduce the mental workload necessary for a human to process the visualization. Since the pathway has already been pruned, a list representation would not removing as much information. Thus, the pruned pathway visualization should be further simplified by restructuring it into a list that contains the pathway’s tasks in an intuitive, natural order.

For the third aspect, we needed a deterministic definition of what we consider a natural order. We decided to use the definition of a topological order from graph theory to find such an order. We used the default notation for graphs in theoretical computer science as, for example, used by Gibbons [34]. A topological order in graph theory is an ordering of the vertices of a directed acyclic graph where for every edge between 2 vertices u and v, the vertex u comes before the vertex v in the ordering.

Obviously, the graph often contains circles. To be able to apply a topological sorting algorithm, we internally remove the last edge from each circle. The last edge is the one that is the farthest from the starting vertex. The resulting order of the vertices, which represent procedures in our case, is one in which the procedures could potentially be executed.

Figure 3 shows a sketch that visualizes how such a context-sensitive visualization should look: given a guideline definition and context information (here: patient data), a pruned list of tasks (with assigned temporal role) can be created. Figure 4 illustrates the intermediate results after each of the 3 processing steps. To summarize, the context-specific guideline visualization provides support for the currently active tasks (point-in-time decisions) by identifying the currently relevant tasks (PRESENT). However, it also offers a simplified orientation over the entire treatment process by also showing past and (potential) future tasks and embedding the currently active tasks between them (perspective decisions).

### System Architecture

**Figure 5:**
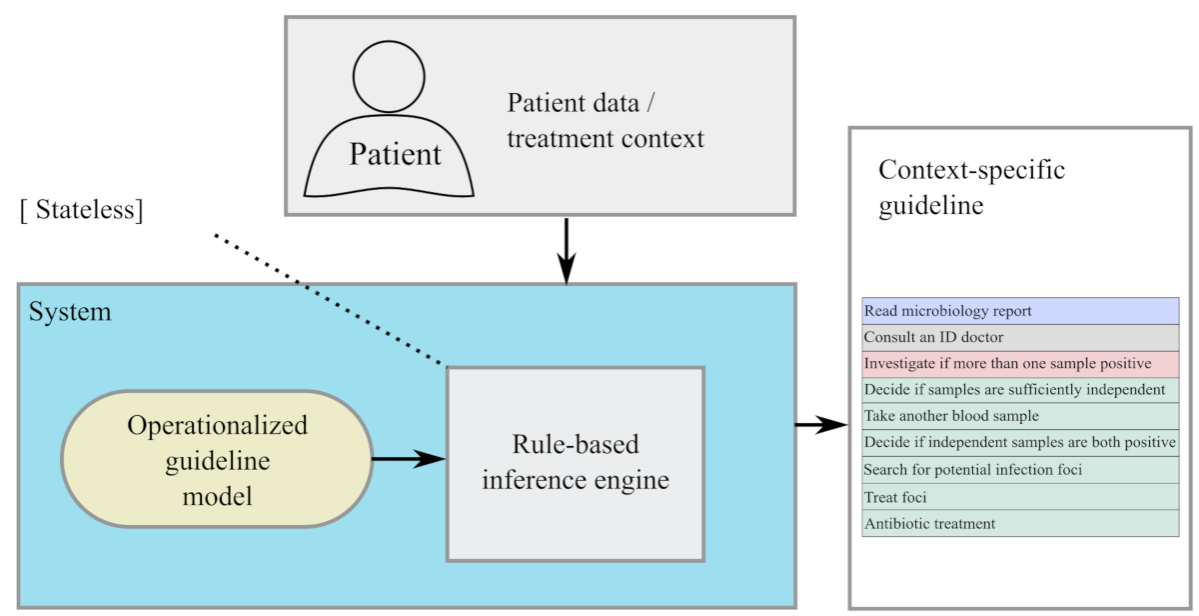
Schematic visualization illustrating stateless guideline processing by the system.

One problem with a treatment context is that a change in 1 parameter can turn the entire state of the clinical pathway upside down or even invalidate it (eg, in critical escalation scenarios). Thus, we designed the system in a stateless way. Each time the context changes (even in a minor way), the entire processing is redone. The system uses the knowledge specified in the operationalized guideline to infer a context-specific visualization each time a treatment context is given (see Figure 5). The prerequisite for this approach is that the full patient history is taken into account for each request. In this way, the contextualized guideline is constructed anew each time. Since the system produces a deterministic result, the context-specific guideline will not change if the change in the context did not have any relevant impact on the guideline interpretation.

### Internal CIG Representation

The CIG is processed by creating an enriched internal graph model. Start events, end events, gateways, and activities are the graph’s vertices. A type property is assigned to each vertex describing which of the BPMN elements this vertex represents. For activities, the respective relevant properties of the treatment context are also stored. Sequence flows (ie, the transition elements of BPMN) are mapped to edges. For each edge, the original traversal condition from the sequence flow is stored.

### CIG Interpretation

#### Use of a Business Rule Engine for Model Interpretation

The internal CIG model is interpreted using a business rule engine, a software system that executes business rules. These rules are typically structured in a “When PREDICATE then CONSEQUENCE” fashion. Business rule engines are closely related to the concept of logical programming. We created a set of business rules that define how the aforementioned internal CIG model shall be interpreted.

In the following sections, we will outline how these business rules are defined to infer a context-specific guideline representation that fulfills the 3 visualization aspects.

#### Pruning and Temporal Assignment

Given a CIG and a patient, the system first infers basic facts about the vertices and edges of the CIG. For example, for each vertex the rule system decides, by applying the patient data, if the activity was already performed or not. For each edge, it is decided if the condition is satisfied, unsatisfied, or cannot be decided with the data provided. These basic facts are then added into the rule system as predicates. The rules are then executed, inferring facts from the known predicates consecutively.

Eventually, a temporal role is inferred for each vertex, determined by the CIG’s logic (gateway logic, conditional logic). Finally, the newly created facts are used to generate the contextualized guideline.

At this point, the contextualized guideline is still a graph, but vertices that are unreachable from the start vertex have already been pruned. While the shape of the context-specific guideline representation is conceptually determined by the 3 aspects already explained, the processing is only separated into 2 steps, since the pruning is implicitly performed during temporal assignment.

#### Topological Sorting

To obtain a linear structure from the graph that can be intuitively understood within the context used, a topological sorting algorithm is applied. The algorithm first removes circles in a way that preserves paths that begin in the starting vertex. Afterward, the algorithm is a modified version of Kahn’s algorithm [35] that keeps vertices close to each other that are close in the original graph.

## Results

### Software Architecture

**Figure 6:**
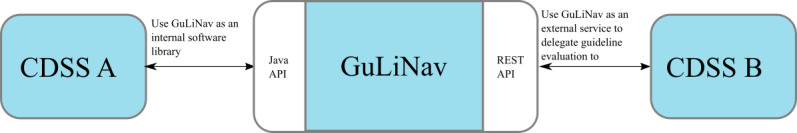
Two distinct interfaces provided by the system: internal software library (Java API) or HTTP (representational state transfer API). CDSS: clinical decision support system; GuLiNav: GuideLine Navigator; API: application programming interface; REST: representational state transfer.

We have created a Java package that provides GuLiNav [36]. The app provides an internal Java application programming interface (API) and can be used as a software library within another Java project. Additionally, GuLiNav can run on its own and provides a representational state transfer (REST)-based interface that can be used to provide guideline models and context information to the system and in return provide context-specific guideline representations (see Figure 6).

**Figure 7:**
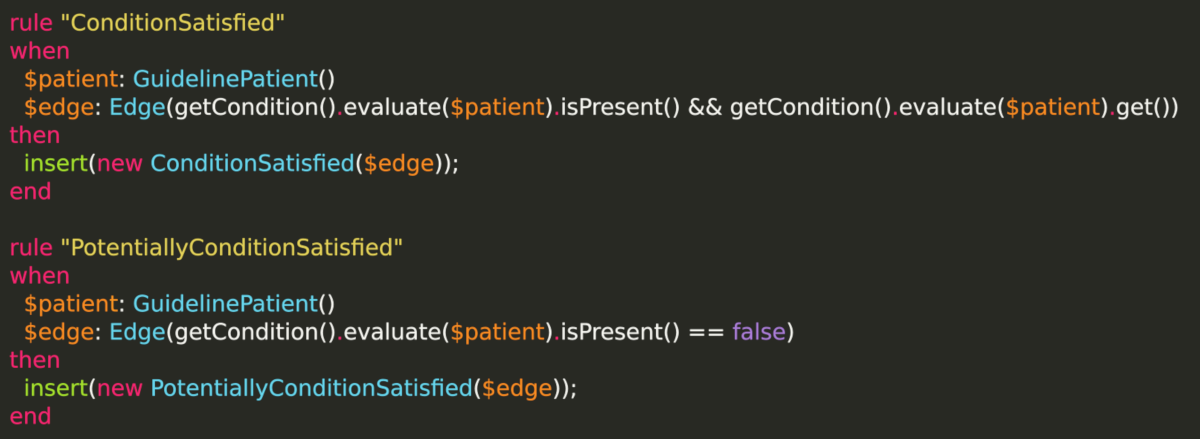
Exemplary Drools rules that are part of the rule system.

The inference of a context-specific representation is performed using a business rule engine. Note that the medical knowledge is not encoded using business rules. Rather, the business rules define how a CIG that is modeled using BPMN should be interpreted. An example can be seen in Figure 7. The criteria to be evaluated in each task can become arbitrarily complex and need to be evaluated in an additional layer that could use, for example, ARDEN syntax [26], but can be provided in arbitrary fashion (we, for example, defined a rather minimalistic software module where medical knowledge is encoded in an ARDEN medical logic modules–like fashion). The structure and purpose of the software facilitates software testing.

### Frameworks Used

We used Spring Boot (2.1.8.RELEASE) [37] to create the REST-API. The rule-based inference is performed using the Drools (7.26.0.Final) [38] business rule engine. The BPMN model is read using the model API of the Camunda engine (7.11.0) [39]. Unified expressions are evaluated using the Java Unified Expression Language library (2.2.7) [40], and unit tests were defined using JUnit (4.4) [41]. For a proof of concept, we have also temporarily embedded the Arden2ByteCode compiler by Gietzelt et al [42] into GuLiNav and defined some exemplary ARDEN medical logic modules and used them for the evaluation of medical criteria.

### Summary of the Concept and Software Implementation

**Figure 8:**
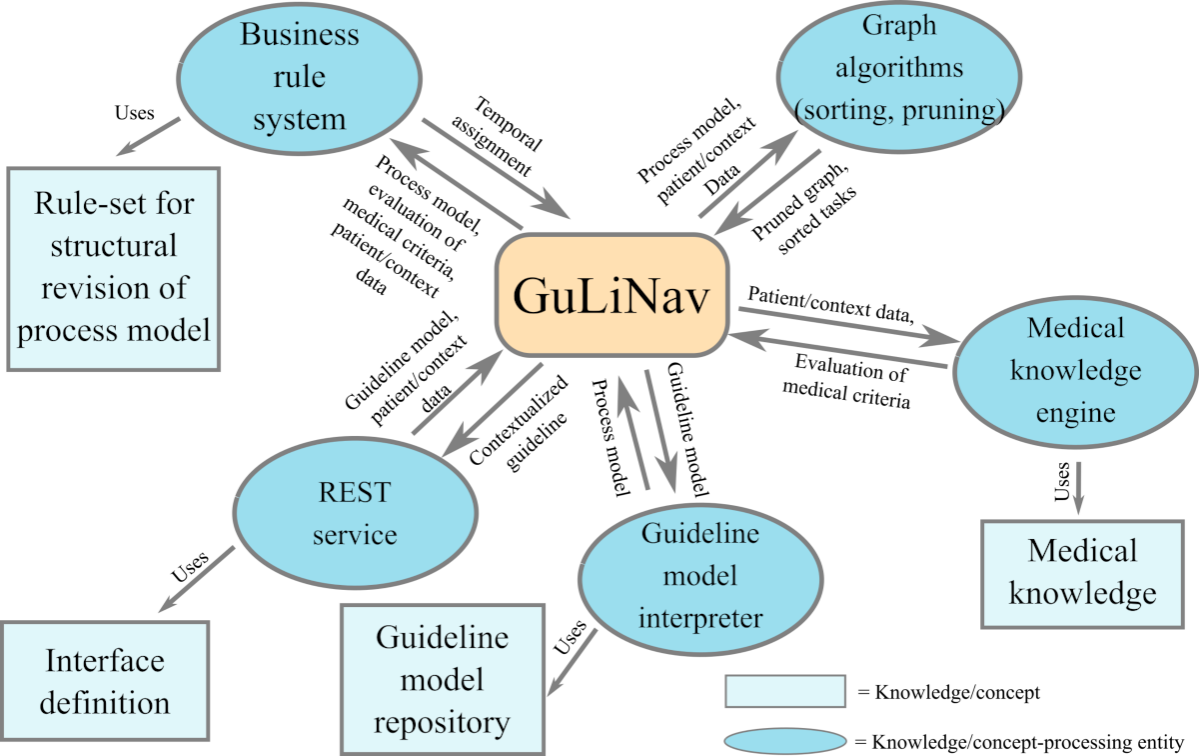
Overarching diagram describing the relation between concepts and technologies used by the GuideLine Navigator. GuLiNav: GuideLine Navigator; REST: representational state transfer.

To provide a summary for the concept and its technical implementation, Figure 8 shows an overarching diagram of different components and concepts used. The central component, GuLiNav, orchestrates the other modules of the system. As a communication point with the external software layers, a REST interface is provided (here: Spring Boot). External programs can use this interface to post new guideline models or request guideline contextualization of a previously posted guideline by posting patient and/or context data. The guideline model interpreter can provide process models of a previously posted guideline by interpreting the corresponding guideline model (here: Camunda BPMN). The medical knowledge engine is responsible for evaluation of the medical criteria layer as described in Fortmann and Spreckelsen [30]. That could be, for example, the aforementioned ARDEN engine, which uses encoded medical knowledge (eg, ARDEN MLMs). Pruning and topological sorting are performed by state-of-the-art graph algorithms directly implemented as part of GuLiNav. Finally, a business rule system (here: Drools) is used to infer the abstract temporal assignment of each of the guideline’s tasks. It uses a rule set that defines how the temporal roles can be inferred from the combined information of the process model, evaluated medical criteria, and patient/context data. GuLiNav then eventually combines all the subsystem’s responses to return a contextualized guideline via the REST interface.

### Knowledge Engineering

Knowledge acquisition for GuLiNav is performed by defining the procedural BPMN model first without completely specifying the medical criteria knowledge required in each task. Since BPMN is a widely adopted standard, there are many modeling tools available, and there is no need to define custom editors. We used Camunda BPMN [39] to model the procedural layer of the CIGs. BPMN is also rather easy to understand for nontechnicians. Thus, these procedural models can be discussed in interdisciplinary teams of information technology specialists and clinicians. The medical knowledge layer of the CIG can also be computerized by computer scientists by discussing individual knowledge modules in natural language with clinicians. These are then transcribed into the self-coded java package directly. The clear separation between procedural knowledge and medical criteria knowledge makes it possible to maintain these parts of the CIG separately, which causes the individual parts to remain relatively simple.

### Demonstrator User Interface

**Figure 9:**
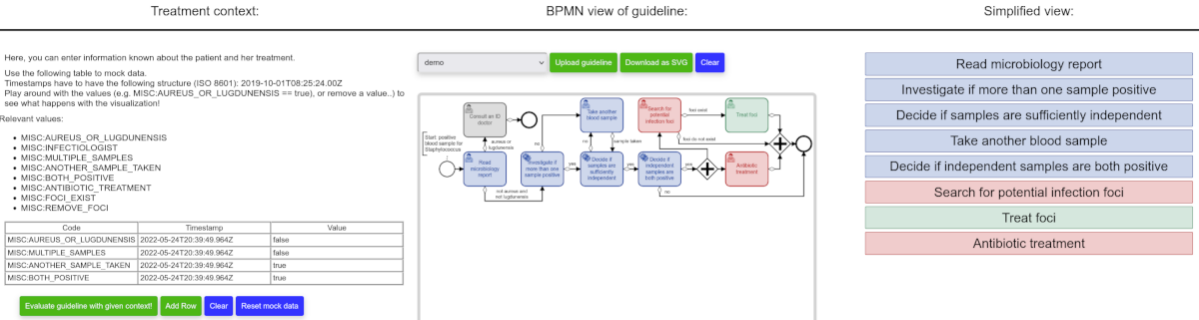
Screenshot of the system’s demonstrator front end.

We created a web-based front end for demonstration purposes. Figure 9 shows a screenshot of the demonstrator. Note that this graphical user interface additionally visualizes an intermediate result (pruned CIG before topological sorting) for debugging and demonstration purposes, which was the original reason for its implementation. This demonstrator can especially be used during interviews with domain experts: changes of the BPMN model can directly be posted to GuLiNav. The impact on the resulting context-specific guideline visualization is then immediately reflected, which enables a direct feedback loop between changes in the procedural model and the resulting context-specific guideline visualization, making it a useful tool for knowledge acquisition.

### Technical Evaluation

**Figure 10:**
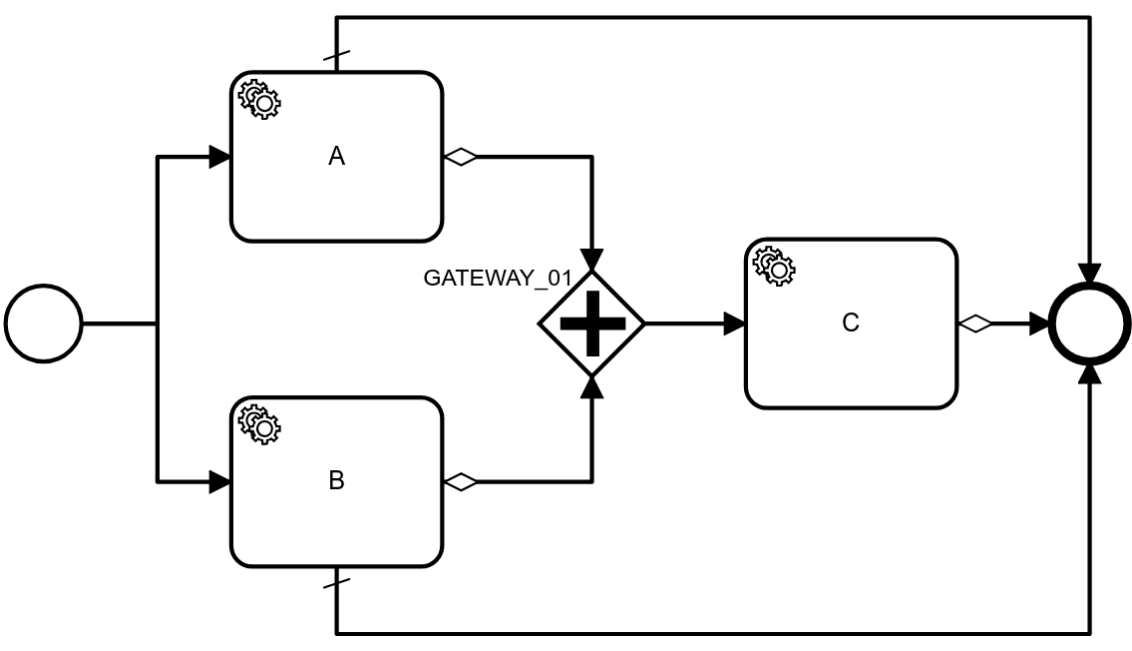
The business process model and notation model used for the test case of the synchronization pattern.

On top of thorough technical testing of GuLiNav using unit tests, we have further validated the inference engine by exploiting the workflow control patterns of Russel et al [23]. Since the semantics of the guideline model are based on those patterns, we could validate the correct behavior of GuLiNav’s inference engine by systematically defining test cases for all supported workflow control patterns. As an example, we present the test case for the synchronization pattern. It is described by Russel et al [23] as “the convergence of 2 or more branches into a single subsequent branch such that the thread of control is passed to the subsequent branch when all input branches have been enabled.” The test case is shown in Table 1, and the pattern itself, modeled in BPMN, is shown in Figure 10. The system processes all possible combinations of patient data for value A and value B and the result is asserted accordingly. Task C does not always have a temporal role because in some cases it is pruned from the contextualized guideline.

**Table 1:** Test case definition for the synchronization pattern.

	Input			Expected inference		
Test-Pat-ID^a^	VAL^b^ A	VAL B	TR^c^ of A		TR of B	TR of C
Pat01	true	true	past		past	present
Pat02	true	—^d^	past		present	future
Pat03	true	false	past		past	—
Pat04	—	true	present		past	future
Pat05	—	—	present		present	future
Pat06	—	false	present		past	—
Pat07	false	true	past		past	—
Pat08	false	—	past		present	—
Pat09	false	false	past		past	—

^a^Test-Pat-ID: test patient ID.

^b^VAL: value for task.

^c^TR: temporal role.

^d^Not present.

### Formative Usability Evaluation

GuLiNav is a framework intended to be used by specific CDSS use cases and as such does not provide a user interface itself. It provides a linear data structure that can be used by the user interface to draw a context-specific guideline just by adding graphical elements. The concrete design in which this guideline representation is shown to the user can thus differ between use cases. We nonetheless executed a formative usability evaluation at this early stage by creating a mobile demonstrator based on the GuLiNav approach, which processes a guideline for the treatment of acute respiratory distress syndrome and presents a context-specific visualization of it to the user. The app was given to 6 clinicians on an iPad and the think-aloud protocol method was used for evaluation [43]. They could manipulate the patient’s data and track the resulting changes in the context-specific guideline visualization. The general feedback toward the mobile app was mixed, and the think-aloud protocol revealed some usability issues. However, the concept of a context-specific guideline visualization in particular was positively received. It was intuitively understood and considered useful by the participants. The complete mobile app was subsequently evaluated using a questionnaire for software ergonomics, but for the context-specific guideline visualization in particular we have, until now, only collected the respective qualitative feedback.

## Discussion

### Principal Findings

GuLiNav shows the technical feasibility of combining CIGs with context information to infer context-specific guideline visualization which avoids cognitive overload while preserving an overview of the global treatment situation. It follows the 3-layer concept [30], in which the procedural aspects of the guideline are encoded using BPMN while the medical knowledge and criteria can be specified using an arbitrary system (eg, ARDEN syntax). It is possible to create such a system as a REST-based service that can then be consulted by other systems whenever needed.

Previous CIG approaches mostly focus on machine interpretability of the structured guideline model with the intention of it being used as a technical component of (multiple) guideline-based CDSSs [14]. They are designed to simplify the software implementation of guideline-based CDSSs and are not intended to be part of the respective system’s user interface. The processing and visualization concept presented in this paper, in contrast, focuses on improving the visualization of the guideline itself. By applying context-specific data to a structured guideline format, it provides a context-specific (human readable) representation. It prevents overwhelming cognitive load and restrictive, inflexible workflows at the same time and thus addresses known issues of CIGs [5,6,8].

It should be noted that the simplifications made when contextualizing a guideline remove procedural knowledge from the original guideline. The result is a linear structure where, for example, no distinction is made between sequential and parallel tasks. Clinicians should generally be able to compensate for this information loss with their implicit knowledge (balance between knowledge in the world and knowledge in the head [44]), but further investigations are necessary to provide evidence. The linear guideline structure is well suited to be displayed on mobile devices with limited screen size. We think this is a major advantage for the development of mobile CDSSs.

### Limitations

This visualization concept is well defined, and clinicians responded positively to it within a formative usability evaluation. However, more detailed evaluations with regard to how well clinicians understand the context-specific guideline depiction (with training or without training) and how much benefit it provides in clinical practice have yet to be performed.

From a technical viewpoint, this implementation demonstrates GuLiNav’s method of operation, but only the most important workflow control patterns (compare with the workflow control patterns as defined by Russel et al [23]) are currently supported for the guideline procedures layer. The medical criteria layer has been implemented as a simple placeholder and is not yet considered in the resulting context-specific visualization.

### Future Work

We intend to use GuLiNav as the back end of (potentially mobile) guideline-based CDSS apps. In the future, the system will be implemented as a generic web service and accessed via a standardized interface. We already began implementing a Fast Healthcare Interoperability Resources (FHIR) interface [45] to provide patient data to the system. The extent to which FHIR’s clinical reasoning module provides the capabilities necessary to potentially also represent the respective static as well as contextualized guidelines in a standardized way has yet to be evaluated.

Even though the 2-way split [30] of the guideline is cleanly separated in the internal implementation of GuLiNav, currently only the procedural layer is presented in the context-specific guideline. This should be addressed in the future, since the guideline knowledge about medical concepts should also be accessible to the end user. One could imagine being able to tap into the items of a context-specific guideline to view the underlying medical concepts. This approach could match well with limited display sizes, which are predominant due to increased use of mobile devices.

Managing the complexity of medical algorithms and guidelines is a core challenge for the successful establishment of guideline-based CDSSs. In addition, existing CDSS-related research projects (such as the study by Hagel et al [9] to use a CDSS for the treatment of bloodstream infections) stressed the need—and critical effort—to obtain regulatory approval as a certified medical device (especially under the Medical Device Regulation). Establishing organizational as well as technical structures to qualify for the respective regulatory approval is inevitably necessary to use the concept in clinical practice. In the future, we plan to use context-specific guideline visualizations within a decision support to be used in clinical practice to evaluate the applicability of the approach in a practical setting.

### Conclusions

Long-term effects and impact on the overarching clinical workflow should be given more attention when working with CDSSs [5]. We approached this proposition by developing GuLiNav, a system that prepares context-specific guideline visualizations aiming at reducing cognitive load while preserving orientation. GuLiNav, in its current form, demonstrates the technical feasibility.

The idea for a contextualized guideline visualization emerged during the early stages of the development of a guideline-based CDSS for the treatment of specific bloodstream infections [9]. The context-specific visualization concept was evaluated as part of a formative usability test, and clinicians generally approved of it. However, further research in actual clinical settings is necessary to better estimate the applicability and usefulness of the approach.

## Abbreviations

API: application programming interface
BPMN: business process and model notation
CDSS: clinical decision support system
CIG: computer-interpretable guideline
CPG: clinical practice guideline
FHIR: Fast Healthcare Interoperability Resources
GLIF3: GuideLine Interchange Format, Version 3
GuLiNav: GuideLine Navigator
HELP: Hospital-Wide Electronic Medical Record Evaluated Computerized Decision Support System to Improve Outcomes of Patients With Staphylococcal Bloodstream Infection
REST: representational state transfer
TNM: task-network model
UML: Unified Modeling Language
